# Use of different but overlapping determinants in a retrovirus receptor accounts for non-reciprocal interference between xenotropic and polytropic murine leukemia viruses

**DOI:** 10.1186/1742-4690-2-76

**Published:** 2005-12-15

**Authors:** Neal S Van Hoeven, A Dusty Miller

**Affiliations:** 1 Division of Human Biology, Fred Hutchinson Cancer Research Center, Seattle, Washington 98109, USA; 2 Molecular and Cellular Biology Program, Fred Hutchinson Cancer Research Center, Seattle, Washington 98109, USA; 3 Current address: Centers for Disease Control, Atlanta, Georgia 30333, USA

## Abstract

**Background:**

Retrovirus infection depends on binding of the retroviral envelope (Env) protein to specific cell-surface protein receptors. Interference, or superinfection resistance, is a frequent consequence of retroviral infection, and occurs when newly-synthesized Env binds to receptor proteins resulting in a block to entry by retroviruses that use the same receptors. Three groups of viruses demonstrate a non-reciprocal pattern of interference (NRI), which requires the existence of both a common receptor utilized by all viruses within the group, and a specific receptor that is used by a subset of viruses. In the case of amphotropic and 10A1 murine leukemia viruses (MLV), the common and specific receptors are the products of two related genes. In the case of avian sarcoma and leukosis virus types B, D, and E, the two receptors are distinct protein products of a single gene. NRI also occurs between xenotropic and polytropic MLV. The common receptor, Xpr1, has been identified, but a specific receptor has yet to be described.

**Results:**

Using chimeric receptor proteins and interference studies, we have identified a region of Xpr1 that is uniquely utilized by xenotropic MLV and show that this receptor domain is required for non-reciprocal interference.

**Conclusion:**

We propose a novel pattern of receptor usage by xenotropic and polytropic MLV to explain the NRI observed between these viruses. We propose that the specific and common receptor determinants for xenotropic and polytropic viruses are simultaneously present in discreet domains of a single Xpr1 protein.

## Background

Retroviral infection of a host cell is initiated by interaction of the retroviral Env protein surface (SU) subunit with a specific host cell receptor. This interaction triggers conformational changes within the Env protein that bring the virus and host cell membranes in close proximity, resulting in fusion and delivery of the viral capsids into the host cell cytoplasm (reviewed in [1,2]). In addition to promoting virus entry, the intracellular interaction of a viral Env and its cognate receptor can limit subsequent infection by subsequent viruses that bind the same receptor. This phenotype is referred to as interference or superinfection resistance because it prevents reinfection of a cell by the same virus strain, and has been used to classify viruses that utilize common cell surface receptors. Currently, mammalian retroviruses are divided into at least 10 different interference groups [3,4]. Within these groups, several retroviruses show a non-reciprocal interference pattern (NRI), where infection by one virus will block infection by a second virus, but infection by the second virus only slightly inhibits infection by the first virus.

As the receptors for different retroviruses have been identified, it has become clear that NRI occurs in cases where related viruses within an interference group utilize a partially overlapping set of receptors for entry. In the case of amphotropic and 10A1 MLV [5] these receptors are Pit1 (Slc20a1) and Pit2 (Slc20a2), the products of two different genes with similar sequence and function. The phosphate transporter Pit2 serves as the receptor for both amphotropic MLV [6,7] and 10A1 [8]. However, 10A1 also binds to the closely related phosphate transporter Pit1, the receptor for gibbon ape leukemia virus (GALV) [9] and feline leukemia virus subtype B (FeLV-B) [10]. Because the amphotropic Env cannot bind to Pit1, it cannot block 10A1 infection of cells that express both receptors, while the 10A1 Env can block amphotropic MLV infection [8].

NRI also occurs among avian sarcoma and leukosis viruses (ASLV) types B, D, and E. Viruses of types B and D can interfere with each other as well as type E viruses, whereas ASLV-E can interfere with itself, but not with types B or D. This group of viruses have all been shown to utilize a common receptor, CAR1 [11,12]. Immunoprecipitation studies with different viral Env proteins have shown that this protein, encoded by the *tv-b *locus in chickens, produces two distinct protein products that differ in their disulfide bond pattern. One form, designated the type 1 receptor, can interact with ASLV-B and ASLV-E, whereas an additional form, the type 2 receptor, is specific for ASLV-B [13].

Another set of retroviruses that show NRI are xenotropic and polytropic MLV (X-MLV and P-MLV, respectively). Studies in cells derived from mink and the wild mouse *Mus dunni *demonstrated NRI between X-MLV and P-MLV [4,14], implying the existence of a common receptor. In both cases, initial infection of cells with X-MLV strains resulted in complete resistance to subsequent infection by P-MLV isolates. However, initial infection of cells with P-MLV strains did not block infection by X-MLV, although the X-MLV titers observed were decreased [4,14]. The hypothesis that these viruses share a common receptor was confirmed by the identification of a single cDNA from humans [15,16] and mice [17] that could mediate infection of both viruses when expressed in resistant cells. However, the identification of a single cell surface receptor is inconsistent with the interference patterns observed between these two viruses. Previously established mechanisms of NRI would suggest the existence of a specific X-MLV receptor that cannot be utilized by P-MLV. Screening of cDNA libraries by three groups independently failed to identify additional genes encoding a xenotropic specific receptor. Furthermore, genetic studies in mice have mapped susceptibility loci for xenotropic and polytropic viruses to the same region of mouse chromosome 1, and it is currently believed that these studies have identified alleles of the same gene [18,19]. These studies collectively argue against the existence of a separate locus encoding an X-MLV specific receptor, and suggest that the specific and the common receptor are encoded by the same gene.

**Figure 1:**
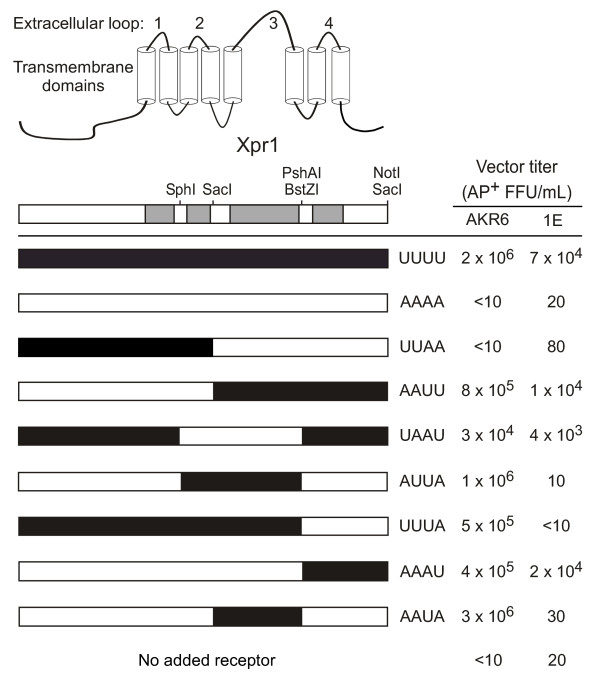
**Analysis of human/hamster Xpr1 chimeras for receptor function**. The predicted transmembrane domain structure of Xpr1 is shown at top and a corresponding block diagram is shown just below with the extracellular loops (ECL) shown in grey. A series of chimeras were constructed by exchange of the indicated fragments of hXpr1 and haXpr1. Restriction enzyme sites used in construction of the Xpr1 chimeras are shown above the block diagram. Chimeric receptors were subcloned into a retroviral expression vector and were transfected into CHO cells. The cells were then grown in medium containing G418 to select for expression of the Neo gene also carried by the expression plasmid. Cells were then exposed to LAPSN vectors bearing either the AKR6 or the 1E Env and the apparent titers of the vectors were determined. Results are means of at least two independent experiments with triplicate determinations in each experiment.

The common receptor, designated Xpr1, is a multiple-pass transmembrane protein of unknown function, although the gene displays a high homology to the *Saccharomyces cerevisiae *Syg1 gene. In yeast, Syg1 is involved in regulation of G-protein mediated signaling [20]. Current topology models predict that the receptor contains four extracellular loops (ECL), and intracellular amino and carboxy termini (Figure 1). Studies subsequent to the identification of the receptor have found residues within the putative third and fourth ECL, at amino acid positions 500 and 582 of the NIH Swiss mouse Xpr1 protein (mXpr1), that are critical for X-MLV receptor function [21]. Due to the ability of P-MLV isolates to utilize mXpr1, a similar set of residues required for P-MLV function were not identified. Our initial studies have focused on examining the determinants for both X-MLV and P-MLV in the same receptor. Making use of chimeras between the functional human and the nonfunctional hamster Xpr1 orthologs, we have identified regions of human Xpr1 that are sufficient to generate functional receptors for xenotropic and polytropic viruses. These studies suggest that two entry determinants are present on Xpr1. One determinant in the putative fourth ECL can be utilized by X-MLV and P-MLV, while a second determinant present in the third ECL can only be used by X-MLV. These results and additional interference studies support a novel model to explain NRI between these two virus types and have identified the xenotropic-specific receptor determinant as a particular domain of Xpr1.

## Results

### Role of the putative third and fourth ECL of Xpr1 in xenotropic and polytropic virus entry

To identify regions of human Xpr1 (hXpr1) that are required for xenotropic and polytropic virus receptor function, chimeric receptors combining coding sequences from hXpr1 and from the non-functional hamster receptor (haXpr1) were made and tested for receptor function following expression in Chinese hamster ovary (CHO) cells (Figure 1). Chimeric receptors were named based on the order of human (U) and hamster (A) sequences that include the putative extracellular domains of the receptor. Because CHO cells can be infected by some X-MLV strains, we used the Env from an X-MLV strain (AKR6) that was unable to mediate transduction of CHO cells even when haXpr1 was overexpressed in the cells (Figure 1, construct AAAA). We also tested the Env from a P-MLV strain (1E) of Friend mink cell focus-forming virus (FrMCF) that mediates only a low rate of transduction of CHO cells overexpressing haXpr1 (Figure 1, construct AAAA). Both Env proteins could mediate relatively efficient transduction of CHO cells expressing hXpr1 (Figure 1, construct UUUU).

CHO cells expressing the Xpr1 chimeras were exposed to xenotropic [LAPSN(AKR6)] or polytropic [LAPSN(1E)] vectors and vector titers were determined (Figure 1). Cells expressing the UUAA chimera were poorly transduced by LAPSN(AKR6) or LAPSN(1E). Conversely, cells expressing the AAUU chimera were transduced at levels only slightly lower than those observed for hXpr1, indicating that the third and fourth loops of hXpr1 are important for both xenotropic and polytropic virus receptor function. Additional analysis of the determinants in this region shows that either the third or the fourth ECL is sufficient for xenotropic virus entry, but that only the fourth ECL can mediate polytropic virus entry. In particular, the AKR6 xenotropic vector could efficiently transduce cells expressing the AAAU or the AAUA chimeras, while the 1E polytropic vector could infect cells expressing the AAAU chimera but not the AAUA chimera.

### Xenotropic and polytropic Env show reciprocal interference on some chimeric receptors

In previous interference studies, infection with a xenotropic virus blocks subsequent infection by viruses bearing either xenotropic or polytropic Env. In contrast, expression of a polytropic Env blocks subsequent infection by other polytropic viruses, but only slightly inhibits xenotropic infection [4,14]. Using our chimeric Xpr1 proteins, we examined the requirement for different regions of Xpr1 in interference between AKR6 and 1E pseudotype vectors.

**Figure 2:**
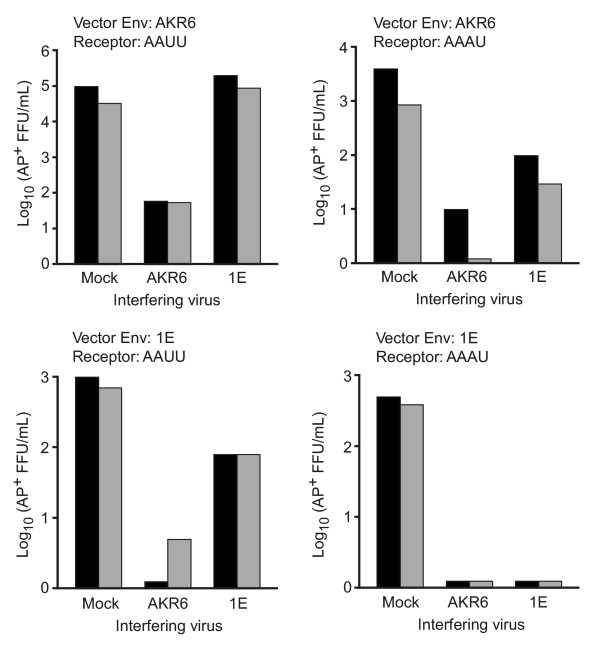
**Analysis of AKR6 and 1E virus interference in CHO cells expressing the AAUU and AAAU chimeric receptors**. CHO cells transduced by retroviral vectors expressing the chimeric receptors AAUU or AAAU were infected with AKR6 or 1E viruses by maintenance of the cells in virus-containing medium or in standard medium (mock infected) for six weeks. After infection the cells were seeded into 6-cm-diameter dishes, were exposed to vectors bearing the indicated Env, and vector titers were determined. Data from two independent infection/vector-titer-measurement experiments, one represented by grey boxes and the other by black boxes, are shown. Titer measurements in each experiment were performed in triplicate.

To establish CHO cell lines expressing both a chimeric Xpr1 receptor and a retroviral Env, CHO cells were transduced with retroviral vectors expressing the chimeric receptors and were then maintained in medium containing replication-competent AKR6 or 1E virus for a period of 6 weeks, as described in Materials and Methods. Cells expressing Xpr1 chimeras and viral Env proteins were challenged with LAPSN(AKR6) or LAPSN(1E) vectors. The level of interference was determined by comparing the titers of LAPSN(AKR6) and LASPN(1E) vectors on mock infected cells versus that on cells infected with a replication competent virus. In CHO cells expressing the AAUU chimera we observed a non-reciprocal pattern of interference between AKR6 and 1E viruses (Figure 2, left panels) similar to that reported previously. Specifically, CHO/AAUU cells infected with AKR6 virus were refractory to transduction by both LAPSN(AKR6) and LAPSN(1E), while CHO/AAUU cells infected with 1E virus were fully susceptible to transduction by LAPSN(AKR6) and were somewhat resistant to transduction by LAPSN(1E). The weak resistance of the 1E-infected CHO/LAAUUSN cells to transduction by LAPSN(1E) is somewhat surprising given that significant levels of interference have previously been described with this Env [4]. The titer we observed was only 10 fold lower than that observed in mock infected CHO/LAAUUSN cells, but was reproduced in multiple independent experiments. Taken together, these results demonstrate NRI for xenotropic and polytropic viruses in CHO cells expressing the AAUU chimeric receptor, similar to that observed previously for xenotropic and polytropic viruses.

The interference patterns on CHO/AAAU cells were markedly different from those described for CHO/AAUU cells. The AAAU receptor contains only a single entry determinant that can be utilized by both AKR6 and 1E pseudotyped viruses. In cells expressing this receptor, transduction by the LAPSN(AKR6) or LAPSN(1E) vectors was blocked by the presence of either AKR6 or 1E Env (Figure 2, right panels), thus showing a pattern of reciprocal interference. Although transduction by LAPSN(AKR6) was not completely blocked by 1E Env, a similar degree of interference was observed in two independent experiments, and the observed differences in titer were found to be statistically significant in both cases by using the Student's t-test (*p* < 0.05).

In summary, these experiments demonstrate a non-reciprocal interference pattern between AKR6 and polytropic viruses on the AAUU chimera, and a reciprocal pattern of interference in the AAAU chimera, which contains only the putative fourth ECL of human Xpr1. These results support the hypothesis that xenotropic virus can utilize either the third or fourth ECL of hXpr1 for cell entry, but that polytropic virus can only use the fourth ECL. When the third ECL is replaced with the non-functional loop from haXpr1, both viruses can only use the fourth ECL for entry and therefore show reciprocal interference.

### SU domains of AKR6 and 1E Env show high sequence similarity to prototypical xenotropic and polytropic Env SU domains

**Figure 3:**
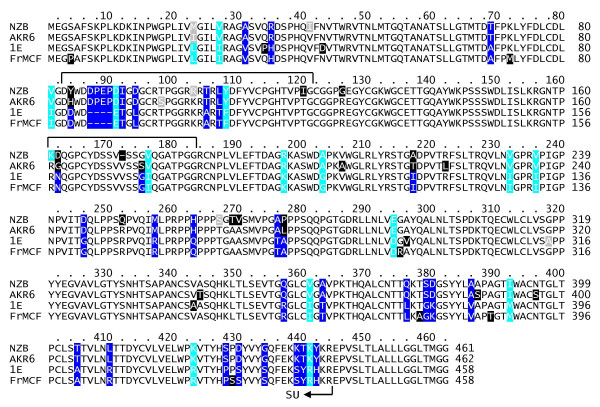
**Amino acid sequence comparison of the Env SU domains of AKR6 X-MLV, 1E P-MLV, and prototypic X-MLV and P-MLV**. Amino acid alignment of the Env SU domains of NZB X-MLV [GenBank:K02730], AKR6 X-MLV [GenBank:DQ199948], 1E P-MLV [GenBank:DQ199949], and FrMCF P-MLV [GenBank:X01679]. Sequences are shown starting with the initiator methionine and include endoplasmic reticulum signal sequences of unknown lengths. Variable regions A and B, believed to be responsible for receptor recognition [45], are indicated by brackets. Non-conservative amino acids differences are indicated by black boxes and conservative changes are indicated by grey boxes. Blue boxes indicate amino acids that are identical among the P-MLVs but dissimilar from one or more of those of the X-MLVs, identical among the X-MLVs but dissimilar from one or more of those of the P-MLVs, or both. Cyan boxes indicate amino acids that are identical among the P-MLVs and similar to those of the X-MLVs, identical among the X-MLVs and similar to those of the P-MLVs, or both.

To characterize the interaction of AKR6 and 1E Env proteins with Xpr1 in more detail, we isolated and cloned the receptor-binding surface (SU) subunits from both proteins. The sequence of the SU region of each Env protein was determined by sequencing a PCR fragment isolated from Hirt DNA extracted from virus-infected dunni cells. Amino acid sequence alignments of AKR6 and 1E SU regions and the those of the prototypic NZB X-MLV [22,23] and FrMCF P-MLV [24] strains shows that the 1E sequence is most like that of the FrMCF virus and the AKR6 sequence is most like that of the NZB sequence (Figure 3). For example, the 1E Env sequence contains a four residue deletion relative to NZB and AKR6 xenotropic Env proteins that is also present in the FrMCF polytropic Env. Additional sequence differences between the Env proteins, many of which occur in two variable regions, are likely to account for differences in host range observed between these viruses.

**Figure 4:**
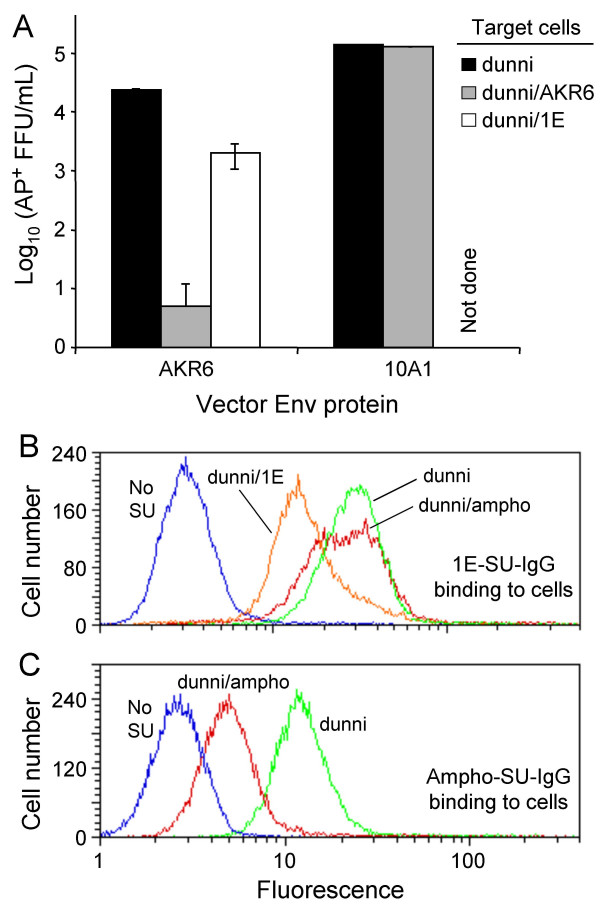
**Binding and interference properties of cloned AKR6 SU and 1E SU**. (A) LAPSN(AKR6env) and LAPSN(10A1env) vector titers were measured on dunni cells and dunni cells infected with replication-competent AKR6 or 1E viruses. Data shown are means ± SD of at least two independent experiments with duplicate determinations in each experiment. (B) Binding of 1E-SU-IgG to dunni cells and to dunni cells infected with replication-competent viruses. (C) Binding of Ampho-SU-IgG to dunni cells infected with 4070A amphotropic virus. Data in (B) and (C) are from a representative experiment and show data from ~18,000 live cells (cells that exclude propidium iodide) per histogram.

A full-length env gene containing the cloned AKR6 SU sequence and the transmembrane (TM) subunit sequence from NZB X-MLV was constructed and was transfected into LGPS/LAPSN cells to generate LAPSN(AKR6env) virus. The titer of this virus on dunni cells was 3 × 10^4 ^AP^+ ^FFU/ml. To verify the identity of the cloned AKR6 Env, we measured the titer of the LAPSN(AKR6env) vector on dunni cells previously infected with replication competent AKR6 or 1E viruses (Figure 4A). LAPSN(AKR6env) transduction of dunni/AKR6 cells was almost completely blocked (<10 AP^+ ^FFU/ml). In contrast, the titer of this vector on dunni/1E cells was reduced by only about 10-fold. As a control, the titer of LAPSN(10A1) vector on dunni and dunni/AKR6 cells was also measured. The 10A1 Env utilizes Pit1 and/or Pit2 for entry, and so should not be affected by the presence of AKR6 xenotropic Env in the cells. As expected, the LAPSN(10A1) titers were equivalent on these cell lines (Figure 4A). The block to LAPSN(AKR6env) transduction in cells chronically infected with AKR6 suggests that the cloned sequence encodes a protein that binds the same receptor as biological isolates of AKR6. Furthermore, the infection patterns observed on dunni/AKR6 and dunni/1E cells are consistent with the NRI previously observed for X-MLV and P-MLV.

A full-length env gene containing the cloned 1E SU sequence and the transmembrane (TM) subunit sequence from NZB X-MLV was constructed and was transfected into LGPS/LAPSN cells, but vector production from these cells was not detected. Examination of multiple 1E-SU PCR clones isolated from various Hirt preparations of 1E virus DNA indicated that the 1E-SU clone we used to construct the Env expression vector does not contain inactivating mutations. Attempts to clone the remaining TM sequence from 1E Env by PCR using primers to conserved regions of Env were unsuccessful, suggesting that 1E may have unique sequences present in the TM domain that are required for proper Env function.

To verify that the cloned 1E SU sequence had the properties of a polytropic virus SU domain, we generated a human IgG tagged version of 1E-SU (1E-SU-IgG). Following production of the protein by transient transfection and purification by FPLC, we examined the binding of 1E-SU-IgG to dunni cells by flow cytometry (Figure 4B). To address the binding specificity of this reagent, and by extension of our cloned SU sequence, we also examined the binding to dunni cells infected with replication competent 1E or with 4070A amphotropic viruses. Similar binding of 1E-SU-IgG was observed in both control and dunni/4070A, whereas reduced binding was observed in dunni/1E cells. As a control, we found that Ampho-SU-IgG protein binding to dunni cells was inhibited in cells infected by an amphotropic virus (Figure 4C). The ability of replication competent 1E virus to inhibit binding of 1E-SU-IgG to cells demonstrates that the cloned SU recognizes a protein that is also bound by the 1E virus isolate. From this result, we conclude that the cloned SU sequence is representative of the Env present in the 1E virus.

### Analysis of xenotropic and polytropic Env binding to cells expressing human, hamster and chimeric receptors

**Figure 5:**
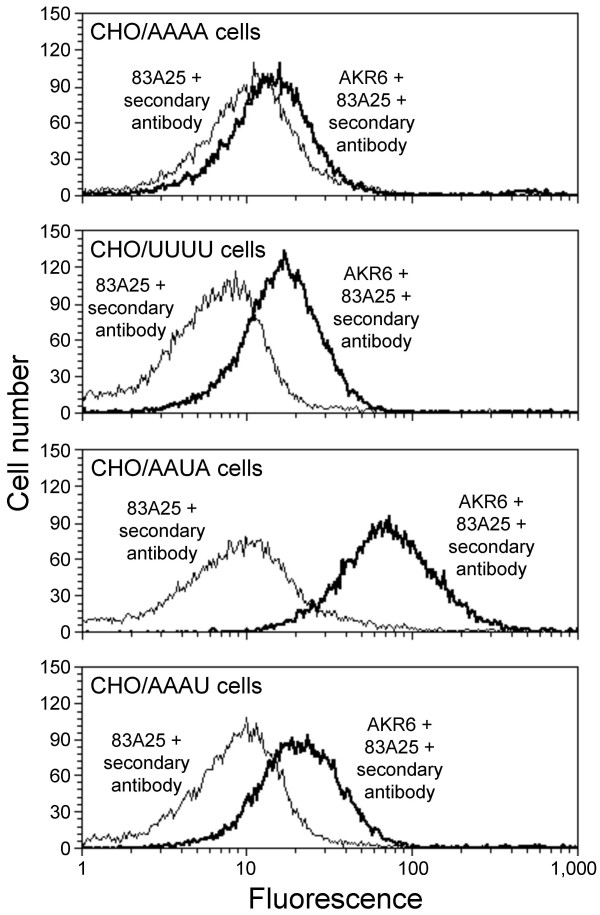
**Measurement of AKR6 virus binding to cells expressing chimeric receptors**. CHO cells transduced with retroviral vectors expressing hamster, human or chimeric Xpr1 receptor proteins were incubated with or without LAPSN(AKR6) virus and virus binding was detected by flow cytometry using the 83A25 anti-Env primary and a fluorescent secondary antibody. Each histogram represents 14,000 to 18,000 live cells (cells that exclude propidium iodide). The experiments were repeated twice with similar results.

The ability of AKR6-pseudotype vector to utilize chimeric receptors that contain either of two non-overlapping regions of hXpr1 suggests that this virus can bind independently to either of the two regions of the cellular receptor. To test this prediction, we measured binding of AKR6 virus to CHO cells expressing various receptors by FACS analysis (Figure 5) using a rat antibody (83A25) that recognizes epitopes in the C-terminus of Env but does not interfere with virus binding to cells [25]. We found a clear increase in AKR6 virus binding to cells expressing hXpr1 in comparison to cells expressing haXpr1. AKR6 virus binding to cells expressing the AAAU chimeric receptor was similar to that of cells expressing hXpr1, consistent with the ability of the AAAU chimera to mediate entry of vectors bearing the AKR6 Env. Interestingly, AKR6 virus binding to cells expressing the AAUA chimera was much higher than that of cells expressing hXpr1. It is important to note that we have not determined the relative cell surface expression levels of the receptors and receptor chimera, and it is possible that differences in binding reflect varied protein levels as opposed to differences in binding affinities. However, binding of the AKR6 virus to cells expressing the AAUA and AAAU chimeras at levels at least as high as to cells expressing hXpr1 is consistent with the hypothesis that the AKR6 Env can independently bind the third or the fourth ECL of hXpr1.

**Figure 6:**
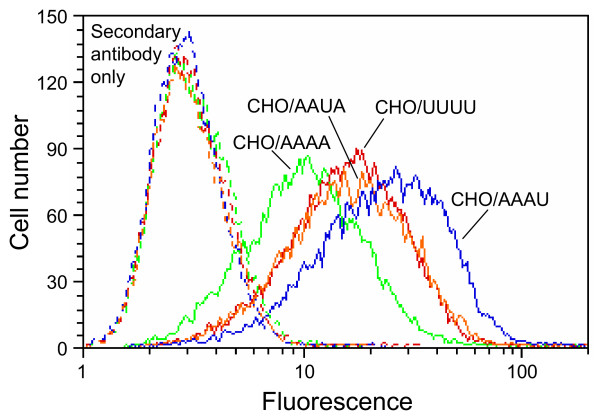
**Measurement of 1E-SU-IgG binding to cells expressing chimeric receptors**. CHO cells transduced with retroviral vectors expressing hamster (AAAA, green), human (UUUU, red) or chimeric (AAUA, orange; AAAU, blue) Xpr1 receptor proteins were incubated with (solid lines) or without (dashed lines) purified 1E-SU-IgG, with fluorescent anti-IgG secondary antibody, and were analyzed by flow cytometry. All analyses were performed in the same experiment with the same FACS settings. Each histogram represents ~13,000 live cells (cells that exclude propidium iodide). The experiment was repeated once with similar results.

The 1E-pseudotype vector could only utilize chimeric receptors that contained the fourth ECL of hXpr1, suggesting that only chimeric receptors containing the fourth ECL of hXpr1 would bind the 1E Env. In this case we could not measure 1E virus binding to cells because the 83A25 rat antibody did not bind to the 1E Env (data not shown), in agreement with previous data showing that 83A25 does not recognize Env from some strains of FrMCF [25]. Instead, to measure 1E Env binding we measured binding of the 1E-SU-IgG protein to cells expressing the chimeric receptors (Figure 6). 1E-SU-IgG binding to hXpr1 was higher than that to haXpr1, consistent with the difference in receptor activities of these proteins. 1E-SU-IgG binding to cells expressing the AAUA chimeric receptor was similar to that for cells expressing hXpr1 while binding to cells expressing the AAAU chimera was higher than that to AAUA- or hXpr1-expressing cells. These results indicate that the 1E Env can bind most efficiently to a receptor containing the fourth ECL (AAAU), but equal binding of 1E Env to AAUA and human Xpr1 was not expected based on the 1E vector transduction data. As with the AKR6 virus binding studies above, it is possible that differences in receptor expression may have influenced these results. In addition, there is relatively high binding of 1E-SU-IgG to haXpr1, a poor receptor for 1E-pseudotype vectors.

## Discussion

Results obtained here with the hamster/human receptor chimeras are consistent with previous studies demonstrating the importance of residues within the putative third and fourth ECL of *Mus dunni *Xpr1 in xenotropic receptor function [21]. In that study, mutations in both the third and fourth ECL of *Mus dunni *Xpr1 were required to abolish xenotropic receptor function while mutations in either ECL alone did not limit virus entry. In the current study, the ability of AKR6 pseudotyped vectors to utilize either the AAUA or the AAAU chimera as a receptor demonstrates that either the third or fourth human ECL is sufficient to support X-MLV entry.

Taken together, our experiments with chimeric receptors suggest a model for entry of X-MLV and P-MLV that is consistent with the NRI observed previously, given that no X-MLV specific receptor has been identified. We propose that two receptor functions are present simultaneously in different domains of Xpr1. One domain, which resides in the fourth ECL functions as a recognition site for both xenotropic and polytropic viruses, while the second receptor domain in the third ECL can only interact efficiently with xenotropic Env.

Our model for NRI predicts that the xenotropic and polytropic viruses should show a reciprocal pattern of interference in a receptor lacking the X-MLV specific receptor domain. The interference experiments described here using the AAAU and AAUU chimeras confirm this prediction. The interference pattern on the AAUU chimera, which contains both entry domains, is non-reciprocal due to the presence of the third extracellular loop. If the xenotropic specific determinant is removed, as in the AAAU chimera, X-MLV entry is markedly inhibited in cells expressing the 1E Env. This finding demonstrates that the third ECL is required for NRI, and that a chimeric receptor lacking this region serves as a common receptor for both P-MLV and X-MLV.

In the interference experiments described here, 1E Env was sometimes unable to completely block infection by a 1E-pseudotype challenge vector (Table 2). Previous work suggests that such incomplete interference may reflect an inherent inability of P-MLV to completely block their cellular receptor. *In vitro *studies specifically examining the mechanism of P-MLV pathogenesis have shown that infection of cells by polytropic/MCF viruses results in accumulation of unintegrated extrachromosomal viral DNA, suggesting that P-MLV are capable of superinfecting cells in culture [26]. This finding is consistent with studies from other oncoretroviral systems showing that pathogenic viral stains can often superinfect cells [27-29]. Given that receptor mediated interference is the primary mechanism by which viruses prevent superinfection, the demonstrated ability of P-MLV to initiate multiple rounds of infection suggests that some polytropic Env proteins are inherently incapable of blocking certain receptors. However, it should be noted that strong interference by polytropic Env proteins can be observed in some cases (Table 2) [4].

It is tempting to speculate that the regions we have identified through our chimera analyses represent the motifs within Xpr1 that are responsible for binding to the viral Env. The critical portions of the molecule are believed to lie outside of the cell, and therefore represent candidates for SU binding domains. However, it is difficult to accurately predict the topology of transmembrane receptors, as was shown in the case of Pit1 and Pit2. Initial predictions of receptor topology were used to design a number of chimeras similar to those described here. Regions within those chimeras were identified that enhanced infection by GALV or amphotropic MLV respectively, and it was suggested that these regions were responsible for virus binding [30-33]. However, recent experiments have provided a new, experimentally verified topology for Pit2 [34], and several of the previously identified critical regions were found to lie on the inner surface of the cell membrane. Therefore, before a specific role can be firmly assigned to the third and fourth ECL of Xpr1, the topology of the protein must be established.

## Conclusion

Results presented here indicate that the non-reciprocal interference between polytropic and xenotropic retroviruses can be explained by a common receptor domain in the putative fourth ECL of Xpr1 and a specific receptor domain for xenotropic virus in the third ECL of the same Xpr1 protein.

## Methods

### Virus and cell line nomenclature

Cell lines containing integrated retroviral vectors are indicated by the name of the cell line, followed by a slash, followed by the name of the integrated vector (e.g. dunni/LAPSN, or CHO/LN). Retroviral vectors in the viral form are described by the vector name followed, in parentheses, by the name of the replication-competent virus or packaging cell line used to produce the vector [e.g. LAPSN(AKR6), LAPSN(PA317)]. Where packaging cell lines have been used, the Gag and Pol proteins are from Moloney murine leukemia virus.

### Cell culture

Chinese hamster ovary (CHO) cells (CHO-K1, ATCC CCL 61) were grown in minimum essential medium-alpha (α-MEM) (Gibco) supplemented with 10% fetal bovine serum (FBS) (Hyclone). All other cell lines were grown in Dulbecco's minimal essential medium (DMEM) (Gibco) supplemented with 10% FBS. CHO cells expressing chimeric receptors were generated by calcium phosphate-mediated transfection of receptor expression constructs. One day post-transfection, cells were trypsinized and seeded at 1:10 dilution into medium containing G418 (750 μg active compound per ml) and were maintained in selection medium for 7 to 10 days. Surviving cells were pooled and utilized in subsequent transduction assays. *Mus dunni *tail fibroblasts (dunni cells), the generation of dunni/LN, dunni/LAPSN, and helper virus-infected derivatives have been described [4]. LGPS/LAPSN cells [35] are a clone of NIH 3T3 cells that express Moloney MLV Gag and Pol proteins and contain the retroviral vector LAPSN [6]. Retrovirus packaging cell lines used included PA317 [36], PD223 [37] and FlyRD [38]. All cells were grown in a 37°C incubator at 10% CO_2 _and 100% relative humidity.

### Chimeric receptor plasmids and retroviral vectors

Receptor chimeras are named to indicate the origin of the sequence in each putative extracellular loop, based on the receptor topology model provided in Figure 1. This model has been suggested in previous studies [21], and was confirmed for this study by using a number of topology prediction algorithms located on the ExPASy proteomics server [39]. For the human/hamster Xpr1 receptor chimeras (Figure 1), "A" indicates sequence from the *Cricetulus griseus *hamster receptor [GenBank:AF198106], while a "U" is used for the human sequence derived from a HeLa cell cDNA library [GenBank:AF099082]. Chimeric Xpr1 proteins were constructed by exchanging restriction fragments as indicated in Figure 1. The 2 kb DNA fragments containing the hXpr1 or haXpr1 coding regions were blunt ended with Klenow and was cloned into *Sma*I digested pBluescript II (Stratagene, La Jolla CA). Following the exchange of fragments required to generate chimeric receptors, all constructs were confirmed by sequencing using primers internal to the receptor sequence. Retroviral vectors expressing the chimeric receptors were made by insertion of 2 kb *Xho*I-*Bam*HI fragments containing the receptor coding regions from pBluescript into the retroviral expression plasmid LXSN [40] after digestion of pLXSN with *Hpa*I and *Bam*HI. Additional retroviral vectors used here included LAPSN [6], which encodes AP and Neo, and LN [40], which encodes Neo.

### Viruses and infection assays

The AKR6 xenotropic and 1E polytropic virus isolates were a kind gift from Bruce Chesebro [14]. LAPSN(AKR6) and LAPSN(1E) retroviral vectors were generated by infecting dunni/LAPSN cells with AKR6 or 1E helper virus, as described previously [4]. LAPSN(AKR6env) and LAPSN(1Eenv) vectors were generated by transfection of pSX2-AKR6env and pSX2-1Eenv into LGPS/LAPSN cells using standard calcium phosphate protocols. Briefly, LGPS/LAPSN cells were plated into 6-cm-diameter culture dishes at 5 × 10^5 ^cells per dish approximately16 h prior to transfection. The following day, 9 μg of the Env expression plasmid was transfected into the cells with 1 μg of pCMV-βgal as a control for transfection efficiency. The following day cells were rinsed with PBS, and incubated with 4 ml culture medium per plate overnight. The conditioned medium was collected, filtered through a 0.45 μm pore-size filter, and was frozen at -80°C. Vector titers were determined by limiting dilution assay on dunni cells. Additional viral vectors, including LAPSN (PA317), LAPSN (PD223), and LAPSN(FlyRD), were obtained by collecting conditioned medium from established producer lines.

Transduction assays in cell lines expressing chimeric receptors were carried out as follows. Approximately 16 h before infection, cell lines were plated at 7 × 10^4 ^cells/well into 6-well (d = 3.4 cm) tissue culture dishes. Immediately prior to infection, medium was changed to include 4 μg/ml Polybrene. Virus was added at appropriate dilutions, and the cells incubated for 48 h to allow expression of the alkaline phosphatase protein from the integrated LAPSN vector. Cells were then fixed in 3.7% formaldehyde in phosphate-buffered saline for 8 min at room temperature. Fixed cells were washed three times with phosphate-buffered saline. Endogenous alkaline phosphatase was inactivated by incubating the cells at 68°C for 1 h. Cells were then stained for alkaline phosphatase activity by incubating the cells over night in AP staining buffer (100 mM Tris pH 8.5, 100 mM NaCl, 50 mM MgCl_2_, 1mg/ml Nitro Blue tetrazolium, 100 μg/ml X-Phos). Transduction events were measured by counting AP^+ ^foci.

### Env cloning

Env SU sequences from the AKR6 [GenBank:DQ199948] and 1E [GenBank:DQ199949] viruses were obtained by PCR from low molecular weight DNA obtained from infected cells. Specifically, dunni cells were plated at 10^5 ^cells in 6-cm-diameter tissue culture dishes. Following overnight incubation, the cells were infected at high multiplicity of infection (~100) with helper virus-containing stocks of LAPSN(AKR6) and LAPSN(1E) in the presence of 4 μg/ml Polybrene (Sigma). 16 h post-infection, low molecular weight DNA was isolated using the method of Hirt [41]. Env sequences corresponding to the SU portion of Env were isolated by PCR using primers Xeno5'env (5'-ATGGAAGGTTCAGCGTTCTCAAAACCCC-3') and Xeno3'Env (5'-TGCCGCCCATAGTAAGTCCTCC-3'). Following gel purification using a Qiaquick gel purification kit (Qiagen), fragments were cloned into pCR2.1 using a Topo-TA cloning strategy (Invitrogen, Carlsbad CA). Full length Env coding regions were generated by ligation of a *Sac*I-*Xho*I fragments into pBS-TM, a pBluescript-based vector containing a C-terminal fragment from the NZB *env *gene [GenBank:K02730]. The pBS-TM plasmid was made by insertion of a *Sac*I-*Not*I fragment from pCSI-ENZB [16] into pBluescript II. Expression plasmids were generated by subcloning of *Xho*I-*Not*I fragments into pCR3.1 (Invitrogen) to generate pCR3.1-AKR6env and pCR3.1-1Eenv. To improve expression in murine and CHO cells, a *Bam*HI-*Hin*cII fragment containing the human cytomegalovirus immediate early promoter was replaced with a *Bam*HI-*Nhe*I fragment containing the Moloney MLV LTR promoter and enhancer from pSX2 [42], to generate pSX2-AKRenv and pSX2-1Eenv. These plasmids were sequenced to confirm the presence of complete Env open reading frames.

The 1E-SU-IgG plasmid was generated by ligation of a *Sac*I-*Xho*I fragment from pCR2.1-1E-Env into pCI-NSU?9-hFc [16]. To confirm the identity and integrity of the resulting fusion protein, the construct was sequenced using primers internal to the 1E-SU.

### Virus and Env SU binding assays

Production and purification of 1E-SU-IgG for binding assays was carried out as described for other similar proteins [43,44]. For flow cytometry assays, 10^6 ^cells were incubated with 1–2 μg of purified fusion protein in a final volume of 100 μl for 2 h. Following washing, cells were incubated with a fluorescent anti human-IgG secondary antibody (DAKO F0315) for 1 h. Cell fluorescence was determined by flow cytometry on a FACSCalibur (BD Biosciences), and data was analyzed using CellQuest software.

For virus binding assays, 10^6 ^cells expressing the indicated receptor chimeras were incubated with LAPSN(AKR6) virus at 4°C for two h. Cells were washed three times with phosphate-buffered saline containing 2% FBS and were incubated with 1 ml hybridoma supernatant containing 83A25 antibody for 2 h. Following two additional washes and incubation with a FITC-conjugated anti-Rat-IgG secondary antibody, cell fluorescence was determined by flow cytometry using a FACSCalibur.

### Interference assays

To establish CHO cell lines expressing high levels of AKR6 and 1E Env, cells were maintained in conditioned medium from dunni/LN cells (mock), or dunni/LN cells productively infected with AKR6 or 1E helper viruses. Conditioned medium (α-MEM with 10% FBS) was collected, centrifuged at 1,000 × g for 10 min to remove cells and debris, and frozen at -80°C for 24 h. Prior to addition to CHO cells, a 1:1 mixture of dunni conditioned medium and fresh α-MEM with 10% FBS was supplemented with 4 μg/ml Polybrene to facilitate infection. The conditioned medium mix was added to cells every 24 h. As CHO cultures reached confluence (approximately every 3 days) cells were removed from the culture dish with trypsin/EDTA and split 1:10 into new 6-cm-diameter dishes. After 6 weeks, cells were trypsinized, counted on a hemacytometer and plated at 10^5 ^cells/well in 6 well dishes. Cells were then transduced with LAPSN(AKR) or LAPSN(1E) viral vectors. The titer of each vector was determined by limiting dilution. The degree of interference can be determined by comparing the vector titer on mock infected cells to that obtained on cells infected with AKR6 or 1E viruses.

## Competing interests

The author(s) declare that they have no competing interests.

## Authors' contributions

NSVH helped design the study, carried out the experiments, analyzed the data, and drafted the manuscript. ADM helped design the study and write the manuscript.
